# Impact of long-term vs. short-term and single day vs. single dose of antibiotic prophylaxis in reducing infection rates after orthognathic surgery: a systematic review and meta-analysis

**DOI:** 10.4317/medoral.26368

**Published:** 2023-12-27

**Authors:** Xiwen Tang, Ke Wen, Yang Yang

**Affiliations:** 1 Department of Stomatology, Liuyang Hospital of Traditional Chinese Medicine, China; 2 Department of Stomatology, Tian He Clinic, China; 3 Department of Orthodontics, Shanxi Dental Hospital, China

## Abstract

**Background:**

This review was designed to examine the effect of long-term (≥2 days) vs. short-term (1 day) and single-day vs. single preoperative doses of antibiotic prophylaxis on surgical site infection (SSI) rates after orthognathic surgery.

**Material and Methods:**

PubMed, Web of Science, Embase, and Scopus were searched for randomized controlled trials (RCTs) without any date or language restriction till 1st September 2023. SSI rates were pooled to generate risk ratio (RR).

**Results:**

Eight RCTs comparing long-term vs. short-term and three RCTs comparing single day vs. single preoperative dose of antibiotic prophylaxis were included. Meta-analysis showed that the use of long-term antibiotic prophylaxis significantly reduced the risk of SSI after orthognathic surgery as compared to short-term antibiotics [RR:0.42 (95% CI: 0.23, 0.76) I2=0%]. Meta-analysis also noted that patients receiving a single day of antibiotic prophylaxis had significantly reduced risk of SSI as compared to those receiving only a preoperative single dose of antibiotics [RR:0.28 (95%: 0.09, 0.82) I2=0%].

**Conclusions:**

Evidence from a limited number of RCTs with moderate to high risk of bias shows that two to seven days of long-term antibiotic prophylaxis reduces the risk of SSI as compared to single-day antibiotic therapy. Also, a single day of antibiotics may be more beneficial than a single pre-operative dose of antibiotic.

** Key words:**Penicillin, antimicrobials, infection, sagittal split osteotomy.

## Introduction

Orthognathic surgery has become the standard procedure for the management of dentofacial deformities involving abnormal positions of the skeletal bases. Le-fort 1 osteotomy and the bilateral sagittal split osteotomy (BSSO) have become the workhorses for repositioning the maxillary and mandibular skeletal bases respectively (1,2). All orthognathic surgeries are clean-contaminated surgeries with infection rates of 10-15% owing to the high microbial load of the oral cavity, nasal cavity, and maxillary sinuses (3). Research has shown that bi-jaw surgery, mandibular surgery, and duration of surgery could be risk factors for surgical site infections (SSI) in patients undergoing orthognathic procedures (4).

Antibiotics have long been used to prevent SSI in orthognathic surgery but there is no consensus on the duration and number of doses required (5). In most cases, orthognathic surgery is performed for cosmetic reasons and is an elective procedure. Hence, surgeons are fearful of SSI which could compromise the final outcomes (6). Furthermore, patients with SSI require prolonged hospital stays and further interventional procedures which could reduce patient satisfaction and increase costs (7). Prolonged doses of antibiotics are often used to limit SSI but at the cost of antibiotic-related adverse effects and anti-microbial resistance (8,9).

Indiscriminate use of antibiotics is often observed in both medical and surgical specialties even when not indicated or in the presence of strong evidence justifying against the use of antibiotics (10-13). Nevertheless, the marked indiscriminate increase in antibiotic use and over-the-counter availability of common antimicrobials has threatened the management of severe infections in critically ill patients. The World Health Organization has identified antibiotic resistance as amongst the three most important public health threats of this century (9).

In this context, high-quality evidence must be obtained on the efficacy and duration of antibiotic therapy for every surgical procedure. Several previous reviews have examined this clinical question but with a limited number of studies resulting in inconclusive evidence (5,7,8,14). Also, past reviews have combined studies comparing long-term vs. short-term and single-day vs. single preoperative doses of antibiotics in the same meta-analysis(8,14). Given the publication of new literature and limitations of the past reviews, we conducted this systematic review and meta-analysis to assess the impact of the duration of antibiotic prophylaxis, i.e. long-term vs. short-term and single day vs. single preoperative dose, in reducing SSI after orthognathic surgery.

## Material and Methods

- Inclusion criteria

This review complied with the PRISMA guidelines (15) with preregistration on PROSPERO. The protocol number allotted was CRD42023447912. The review questions were two 1) “Is there a difference in the risk of SSI with long-term or short-term antibiotic prophylaxis in patients undergoing orthognathic surgery?” 2) is there a difference in the risk of SSI with single-day or single-dose antibiotic prophylaxis in patients undergoing orthognathic surgery?”

Consistent with this question, the inclusion criteria were formulated by observing the PICOS criteria. We included studies fulfilling the following:

Population: Conducted on patients undergoing any type of orthognathic surgery

Intervention: 1) Receiving a long-term dose of any antibiotic post-surgery (≥2 days) 2) Receiving a single-day dose of any antibiotic post-surgery.

Comparison: 2) Receiving a short-term dose of the same antibiotic post-surgery (1 day) 2) Receiving a single preoperative dose of any antibiotic post-surgery.

Outcome: SSI

Study type: RCTs only.

Retrospective studies, single-arm studies, trials comparing antibiotics with placebo, and not specifically on orthognathic surgery patients were excluded. Similarly, non-peer review studies, unpublished data, and editorials were not considered.

- Search

The trials pertinent to the review were searched online by two reviewers. The scanned databases included PubMed, Web of Science, Embase, and Scopus. No date or language restriction was applied with the search culminating on 1st September 2023. The keywords of the search consisted of: “le-fort 1 osteotomy”; “bilateral sagittal split osteotomy”; “BSSO”; “orthognathic surgery”, “antimicrobials”, and “antibiotics”. A search string was generated combining these keywords with AND and OR. The same two reviewers pooled all articles obtained from the databases into a deduplication software to eliminate the same studies. The unique list of articles was then screened based on the above-defined inclusion criteria. First title and abstract screening was done followed by a full-text review. The third reviewer was called for deliberation and reaching a consensus in case of an inconsistency in study selection. Additional studies were recognized by reviewing the reference lists of previous reviews and included trials.

- Extracted data and study quality

Information was extracted by two reviewers separately and consisted of details on the name of the primary author, year of publication, study location, antibiotic protocol, type of antibiotic and its dose and timing, control group protocol, sample size, age, male gender in the sample, diagnosis of SSI and SSI rates. Study details were checked again by the primary article in case of discrepancies in data collection.

Methodology and risk of bias in every RCT were assessed by the Cochrane Collaboration risk of bias-2 tool(16). Trials were judged for risk of bias on the standard domains of the tool which consisted of the randomization process, deviation from intended intervention, missing outcome data, measurement of outcomes, and selection of reported results. An overall assessment of the risk of bias was then made based on the results of individual domains.

- Statistical analysis

Quantitative synthesis was carried out by “Review Manager” (RevMan, version 5.3; Nordic Cochrane Centre (Cochrane Collaboration), Copenhagen, Denmark; 2014). SSI were reported as dichotomous outcomes and hence pooled using risk ratio (RR) and 95% confidence intervals (CI). Forest plots were produced in the software by using the random-effect meta-analysis model. Between studies, heterogeneity was examined by I2 statistic with a value of >50% meaning substantial heterogeneity. Publication bias was examined using funnel plots. We performed a sensitivity analysis as well to examine if the results changed on the removal of any study. This was done if the meta-analysis had more than three studies.

## Results

- Search results

**Figure 1:**
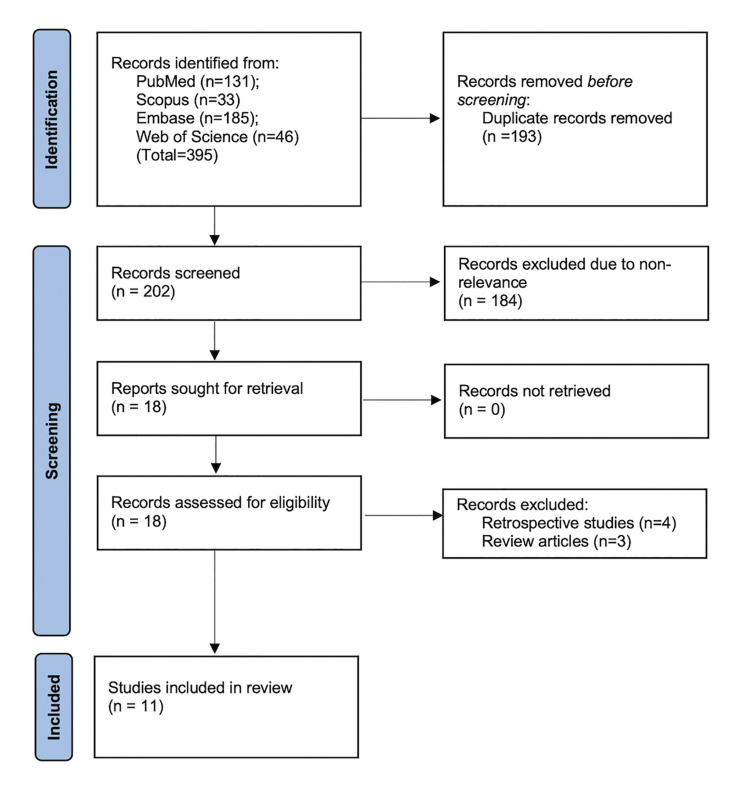
Study flow chart.

A combined search of the three databases retrieved 395 articles. Duplicates were removed and 202 articles underwent screening. Of the 18 selected for full-text review, 11 RCTs were included (17,18,19-27) (Fig. 1).

There were 8 RCTs comparing long-term vs. short-term antibiotic prophylaxis for orthognathic surgery (Table 1). These were conducted in the USA, Canada, Israel, South Korea, Jordan and Thailand between 1984 to 2019. Most studies included all orthognathic surgery patients. Three trials used penicillin G, and one trial each used amoxicillin, amoxicillin-clavulanate, cefpiramide, and cefazolin. One trial had two groups of penicillin G and amoxicillin-clavulanate, data of which was pooled separately. In all studies, clindamycin was the drug of choice if patients were allergic to penicillin. The long-term antibiotic group received 2 to 7 days of antibiotic while the short-term group received 1 day of antibiotic.

**Table 1:**
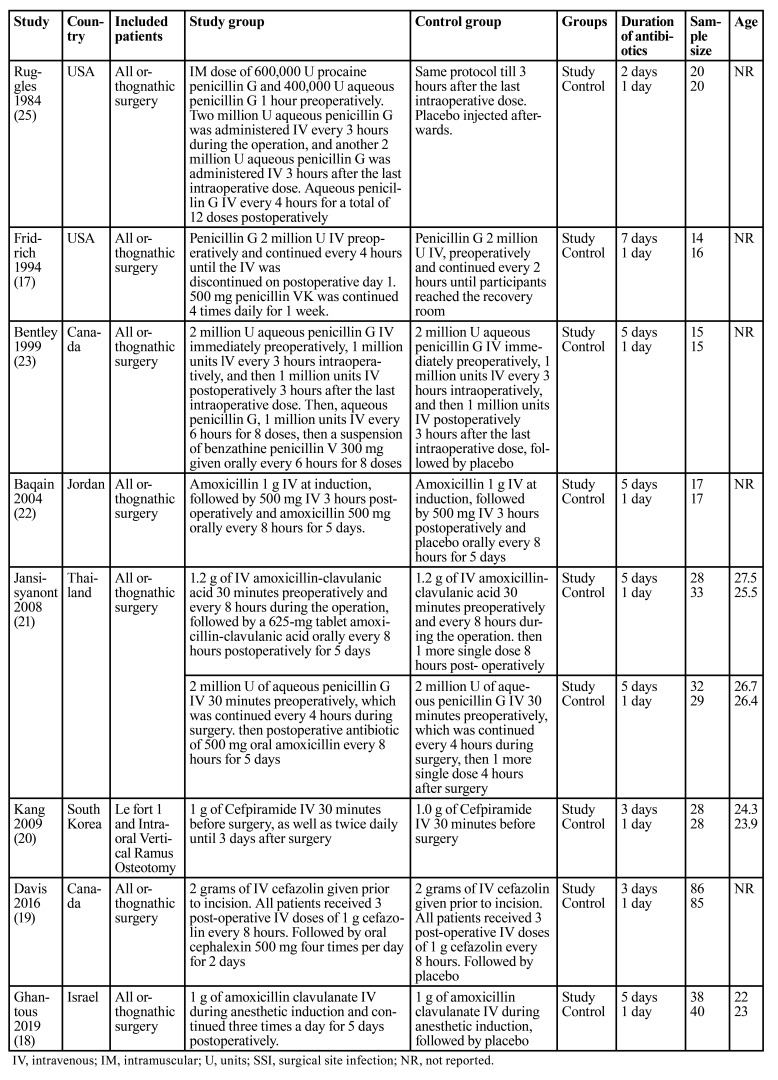
Meta-analysis of SSI between long-term vs. short term and single-day vs single dose antibiotics after orthognathic surgery.

Three trials compared a one-day antibiotic regimen vs. single-dose antibiotic prophylaxis for orthognathic surgery (Table 2). Two trials were from India and one from the Netherlands. The antibiotics used were clindamycin, ampicillin and amoxicillin. Two trials were only on BSSO while one trial included all orthognathic surgery patients. The diagnostic criteria used for SSI in all studies are shown in Table 3.

**Figure 2:**
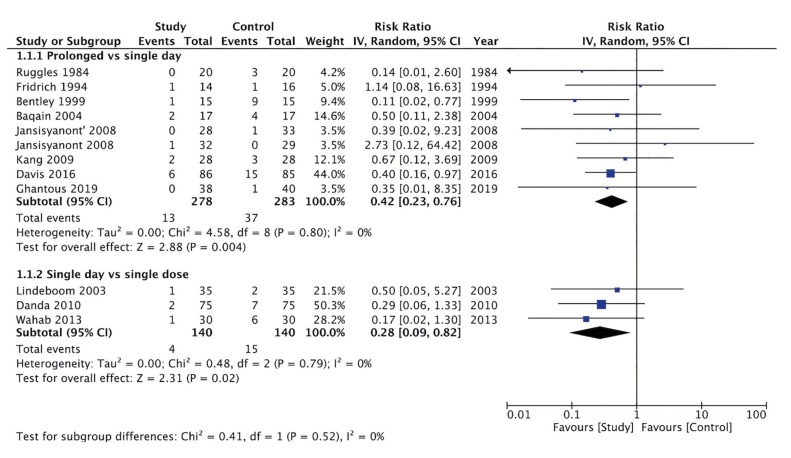
Meta-analysis of SSI between long-term vs. short term and single-day vs single dose antibiotics after orthognathic surgery.

**Figure 3:**
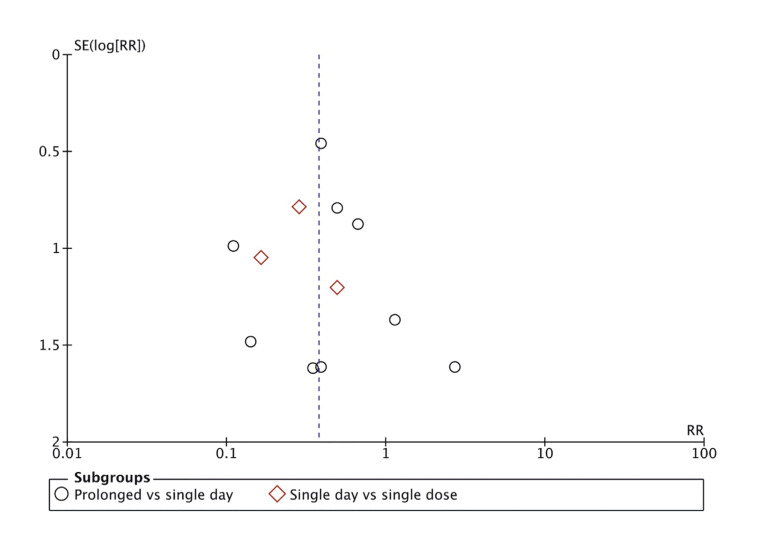
Funnel plot for publication bias.

Meta-analysis of eight RCTs showed that the use of long-term antibiotic prophylaxis significantly reduced the risk of SSI after orthognathic surgery as compared to short-term antibiotics. The overall effect size was RR:0.42 (95% CI: 0.23, 0.76) with I2=0% indicating no interstudy heterogeneity (Fig. 2). The results also failed to change in significance on the removal of individual RCTs. The meta-analysis also noted that patients receiving a single day of antibiotic prophylaxis had a significantly reduced risk of SSI as compared to those receiving only a preoperative single dose of antibiotics. In this case, the effect size was RR:0.28 (95%: 0.09, 0.82) with I2=0% indicating no interstudy heterogeneity (Fig. 2). Publication bias was not identifiable on the funnel plot (Fig. 3).

The author's judgement on the risk of bias in the 11 RCTs is shown in Table 4. We noted that there was only one RCT with a low risk of bias. All others had some concerns or a high risk of bias.

**Table 2:**
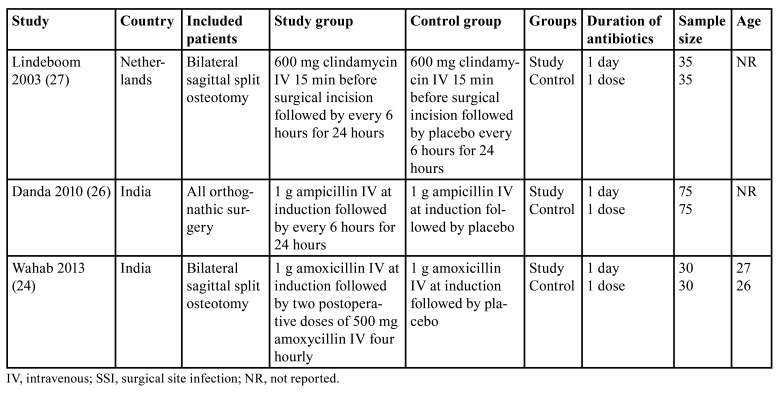
Details of included studies comparing single day vs single dose antibiotic prophylaxis after orthognathic surgery.

**Table 3:**
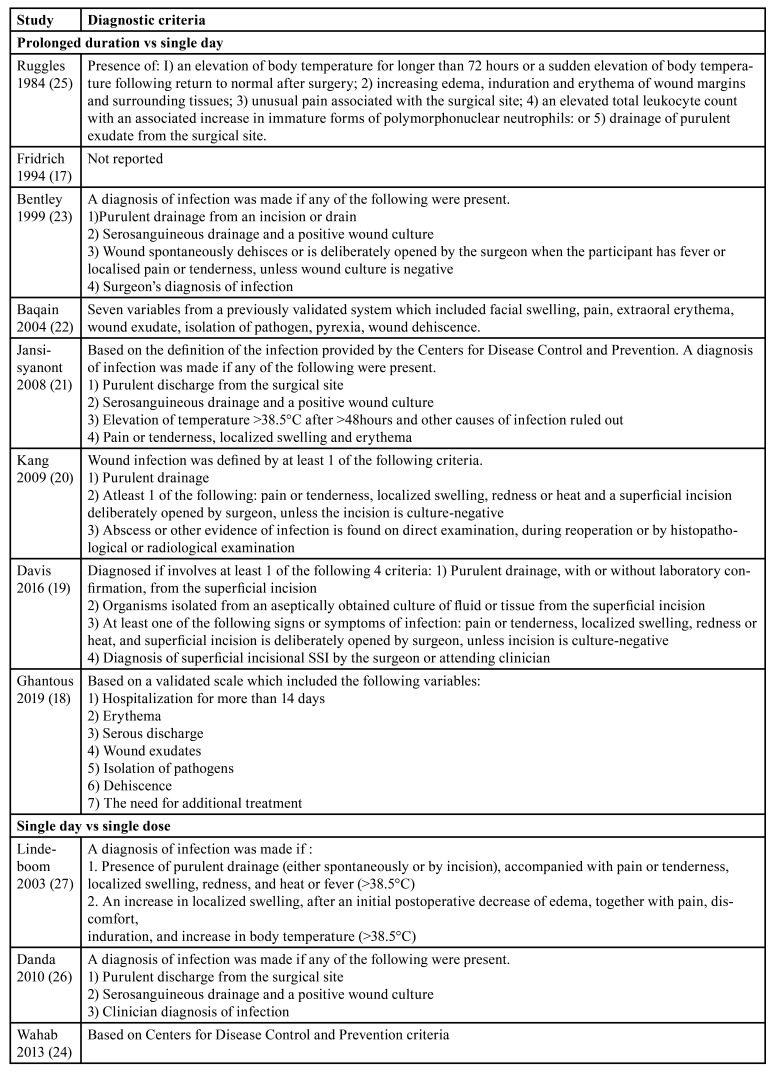
Diagnostic criteria of SSI in included studies.

**Table 4:**
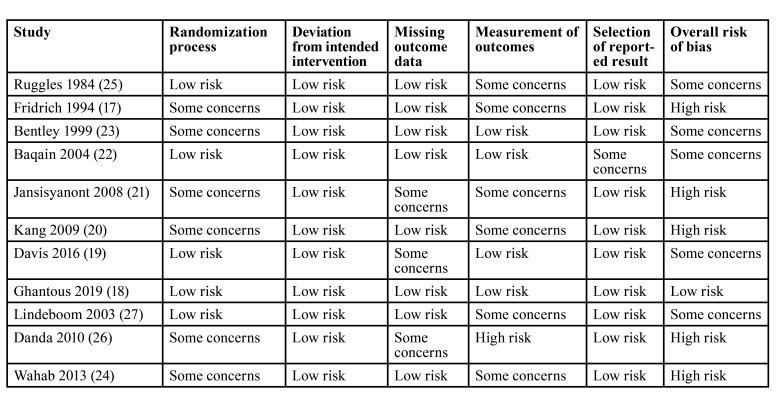
Risk of bias analysis.

## Discussion

The objective of this systematic review and meta-analysis was to generate level 1 evidence on the duration of antibiotic therapy required to prevent SSI after orthognathic surgeries. Therefore, restricting our inclusion criteria to only RCTs but without any publication time limits, we were able to search 11 RCTs providing evidence on the same. The studies were divided into two groups with separate meta-analyses to reduce inter-study heterogeneity owing to the vastly different antibiotic duration protocols. The first group included eight RCTs which compared the duration of postoperative antibiotic therapy (long-term vs. short-term) while the second group which included three RCTs examined if a single day of antibiotics was better than a single pre-operative dose of antibiotic.

Examining the results of the first meta-analysis, we found that two to seven days of post-operative antibiotic therapy significantly reduced the risk of SSI by 56% as compared to a single dose of antibiotic therapy. There was no interstudy heterogeneity noted in the meta-analysis and even the results did not change on removal of individual studies thereby demonstrating the robustness of the effect size. The second meta-analysis showed that as compared to a single preoperative dose of antibiotic, a single day administration of antibiotic significantly reduces the risk of SSI by 72%. However, the results should be interpreted with caution owing to the low number of RCTs available. Amalgamating the two meta-analyses, the review shows that in patients undergoing orthognathic surgeries, a longer duration of antibiotic protocol is more beneficial as compared to single-day antibiotic therapy which in turn is better than a single dose of preoperative antibiotic in reducing SSI.

In comparison with the present review, prior reviews have generated mixed results and have important limitations. Lu *et al* (28) in 2023 published a meta-analysis on the same subject but ended up combining RCTs with retrospective studies and those with long-term vs. short-term and single-day vs. single-dose antibiotic therapy. Furthermore, one trial (29) on facial fracture was also erroneously included in the review which significantly reduces the reliability of the meta-analysis. Danda *et al* (8) in 2011 combined data from eight RCTs to show that extended antibiotic regimen doses have a role in reducing SSI in orthognathic surgeries. Contrastingly, Tan *et al* (14) in the same year reviewed 5 RCTs to note no difference in the risk of SSI between short-term and long-term antibiotic protocols. By incorporating additional RCTs and separating them based on the duration of antibiotics, we believe that the current review presents the best evidence on the topic to date.

Retrospective studies have also generated mixed results on the efficacy of postoperative and long-term antibiotics after orthognathic surgery. A large Japanese study of 181 patients has shown that a shorter duration of postoperative antibiotic therapy (≤3 days) was an independent risk factor for SSI (30). Contrastingly, Peleg *et al* (31) compared 209 orthognathic surgery patients by dividing them into three groups based on the duration of postoperative antibiotic therapy (24 hours, 2-3 days, and >3 days), only to note no difference in the risk of SSI in the three groups. Gaal *et al* (32) in a retrospective review of 333 patients assessed if additional postoperative antibiotics to intraoperative antibiotics reduced SSI after orthognathic surgery. SSI rate was 17.1% in the postoperative antibiotic group and 26.5% in the intraoperative antibiotic group with no statistically significant difference. However, a 2023 study has shown that a single dose of antibiotic is as effective as a 5-day postoperative therapy for preventing SSI in orthognathic surgery (33). While retrospective studies do provide insights into the real-world evidence on antibiotic therapy, their results should be interpreted with a high degree of caution. Selection bias, lack of standardization of antibiotic protocols, and a large number of confounding factors make their results less reliable.

The risk of SSI after orthognathic surgery results in a complex interaction between intra-operative microbial inoculation and the patient's local and systemic resistance to infection (34). In clinical practice, the decision to administer prolonged antibiotics is dependent on several factors like the patient's age, general health, number of surgical sites, duration of the surgery, use of hardware or grafts, and even the surgeon's preference (14). In the current review, we noted no inter-study heterogeneity in the meta-analysis, but there were many methodological variations which need to be considered. The trials incorporated a mix of orthognathic surgical procedures and vastly different antibiotic regimens. It is known that the mandible has a poorer blood supply as compared to the maxilla and gravity causes stagnation of microbiota-rich saliva at the mandibular surgical site which can alter the risk of SSI (14). The selection of antibiotic should be based on the most prevalent pathogen at the surgical site, lack of antibiotic resistance to the antibiotic, and the dose should achieve adequate drug levels before and during the procedure (35). All trials used penicillin or cephalosporins which are broad spectrum and effective against oral pathogens and the choice of antibiotic may reflect the local drug policy based on antibiotic sensitivity. Importantly, none of the included trials reported adverse events associated with prolonged antibiotic therapy. Penicillin’s are associated with gastric adverse events and severe allergic reactions (17,25). Either, there were no adverse events with longer antibiotic therapy in all trials or such data was not reported.

- Limitation

There are other important limitations to the review. Despite being an updated study, the low number of RCTs especially in the second group lowers the confidence of the results. Some of the trials were conducted more than 20 years ago. Changes in antibiotic protocols, the sensitivity of pathogens, refinements of surgical techniques and operation theatre protocols have changed the risk of SSI in the past two decades. Secondly, there were differences in the duration of long-term antibiotic prophylaxis which ranged from two to seven days. Due to a small number of studies, a subgroup analysis was not possible. Thirdly, we were unable to discern SSI with specific orthognathic surgical procedures as most studies included a mix of procedures. Lastly, the quality of evidence was not high as many studies had a high risk of bias and there was only one high-quality RCT.

## Conclusions

Evidence from a limited number of RCTs with moderate to high risk of bias shows that two to seven days of long-term antibiotic prophylaxis reduces the risk of SSI as compared to single-day antibiotic therapy. Also, a single day of antibiotics may be more beneficial than a single pre-operative dose of antibiotic. Further high-quality and large RCTs are needed to enhance current evidence.
